# Differential Abnormality in Functional Connectivity Density in Preclinical and Early-Stage Alzheimer's Disease

**DOI:** 10.3389/fnagi.2022.879836

**Published:** 2022-05-25

**Authors:** Yu Song, Huimin Wu, Shanshan Chen, Honglin Ge, Zheng Yan, Chen Xue, Wenzhang Qi, Qianqian Yuan, Xuhong Liang, Xingjian Lin, Jiu Chen

**Affiliations:** ^1^Department of Neurology, The Affiliated Brain Hospital of Nanjing Medical University, Nanjing, China; ^2^Institute of Neuropsychiatry, The Affiliated Brain Hospital of Nanjing Medical University, Nanjing, China; ^3^Institute of Brain Functional Imaging, Nanjing Medical University, Nanjing, China; ^4^Department of Radiology, The Affiliated Brain Hospital of Nanjing Medical University, Nanjing, China

**Keywords:** subjective cognitive decline, amnestic mild cognitive impairment, functional connectivity density, functional connectivity, functional magnetic resonance imaging

## Abstract

**Background:**

Both subjective cognitive decline (SCD) and amnestic mild cognitive impairment (aMCI) have a high risk of progression to Alzheimer's disease (AD). While most of the available evidence described changes in functional connectivity (FC) in SCD and aMCI, there was no confirmation of changes in functional connectivity density (FCD) that have not been confirmed. Therefore, the purpose of this study was to investigate the specific alterations in resting-state FCD in SCD and aMCI and further assess the extent to which these changes can distinguish the preclinical and early-stage AD.

**Methods:**

A total of 57 patients with SCD, 59 patients with aMCI, and 78 healthy controls (HC) were included. The global FCD, local FCD, and long-range FCD were calculated for each voxel to identify brain regions with significant FCD alterations. The brain regions with abnormal FCD were then used as regions of interest for FC analysis. In addition, we calculated correlations between neuroimaging alterations and cognitive function and performed receiver-operating characteristic analyses to assess the diagnostic effect of the FCD and FC alterations on SCD and aMCI.

**Results:**

FCD mapping revealed significantly increased global FCD in the left parahippocampal gyrus (PHG.L) and increased long-range FCD in the left hippocampus for patients with SCD when compared to HCs. However, when compared to SCD, patients with aMCI showed significantly decreased global FCD and long-range FCD in the PHG.L. The follow-up FC analysis further revealed significant variations between the PHG.L and the occipital lobe in patients with SCD and aMCI. In addition, patients with SCD also presented significant changes in FC between the left hippocampus, the left cerebellum anterior lobe, and the inferior temporal gyrus. Moreover, changes in abnormal indicators in the SCD and aMCI groups were significantly associated with cognitive function. Finally, combining FCD and FC abnormalities allowed for a more precise differentiation of the clinical stages.

**Conclusion:**

To our knowledge, this study is the first to investigate specific alterations in FCD and FC for both patients with SCD and aMCI and confirms differential abnormalities that can serve as potential imaging markers for preclinical and early-stage Alzheimer's disease (AD). Also, it adds a new dimension of understanding to the diagnosis of SCD and aMCI as well as the evaluation of disease progression.

## Introduction

Subjective cognitive decline (SCD) and amnestic mild cognitive impairment (aMCI) have been recognized as the preclinical and prodromal stages of Alzheimer's disease (AD), respectively (Rabin and Smart, 2017; Hadjichrysanthou et al., 2020). SCD is used to describe the condition of elderly people who perceive their cognitive function to be impaired despite the absence of verifiable neuropsychological dysfunction, whereas aMCI refers to individuals who experience memory loss with or without a decline in other cognitive abilities (Xue et al., 2021). Accumulating evidence has suggested that both of them have a high probability of progressing to AD (Rabin and Smart, 2017; Hadjichrysanthou et al., 2020). As for the lack of effective treatment for AD, sensitive diagnostic procedures and prompt interventions are critical.

Resting-state fMRI (rs-fMRI), known as a non-invasive technique, is extensively applied in the AD-related spectrum for detecting functional activity in the brain by measuring various indicators such as functional connectivity (FC) and others (Xu et al., 2020; Song et al., 2021). Functional abnormalities of cortical or subcortical hubs are closely associated with cognitive dysfunction (Yu et al., 2017). Wang et al. discovered altered intrinsic FC patterns with insular subnetworks in patients with SCD and aMCI, as opposed to healthy controls (HCs), and those specific changes were correlated with episodic memory (EM; Wang et al., 2021). Furthermore, using graph analysis, patients with AD or mild cognitive impairment (MCI) had global and local FC disruptions (Zhao et al., 2012; Wang et al., 2013). Abnormal FC between regions with a greater physical distance should also be attenuated. Studies confirmed that long-distance connectivity loss was linked to brain network dysfuction (Liu et al., 2014; Tao et al., 2020). In this investigation, we chose functional connectivity density (FCD) mapping, an ultrafast data-driven method, to identify functional hubs in patients with SCD and aMCI, as it performs multi-perspective assessments of the whole brain, short- and long-range aspects.

FCD mapping quantifies the importance of a voxel by comparing it to all other voxels in the whole brain. The higher a voxel's FCD value, the greater the number of effective FCs it possesses in comparison to other voxels, implying that it is essential for function maintenance (Tomasi, 2010; Tomasi and Shokri-Kojori, 2016). FCD can be further subdivided into global FCD (gFCD), local FCD (lFCD), and long- range FCD (lrFCD) based on neighbor relationships between voxels (Li et al., 2018a). The gFCD of a voxel reflects functional coupling throughout the brain, whereas the lFCD presents local changes, and the lrFCD presents functional integration between voxels that are not adjacent to each other (Tomasi, 2010). Different metrics can describe different characteristics of a functional hub (Tomasi, 2010), and multiscale assessment can be more accurate in disease identification (Liu et al., 2018; Xue et al., 2021). Previous studies have shown dramatic changes in FCD in patients with neuropsychological diseases (Zhuo et al., 2014; Zhang et al., 2015, 2016). Mao et al., while analyzing FCD in patients with AD and MCI, found a disrupted balance of the lFCD and lrFCD in both of them (Mao et al., 2021). However, to the best of our knowledge, no study exploring the alterations of FCD in patients with SCD or aMCI has been conducted.

The purpose of this study was to comprehensively characterize abnormalities of functional coupling in SCD, aMCI, and HC by combining FCD analyses and seed-based FC analyses, as the FCD analysis does not reveal specific regions abnormally connected to the region with altered FCD (Hu et al., 2017). Besides, correlation analyses and receiver-operating characteristic (ROC) analyses were simultaneously performed. We hypothesized that different altered patterns of FCD and FC were shown in patients with SCD and aMCI. These changes may contribute to cognitive deterioration and can help distinguish different phases of preclinical and early-stage AD.

## Materials and Methods

### NBH-ADsnp Database

The data for the applied research were obtained from our domestic database, the Nanjing Brain Hospital Alzheimer's Disease Spectrum Neuroimaging Project (NBH-ADsnp) (Nanjing, China; Chen et al., 2020; Wang et al., 2021; Xu et al., 2021), which is regularly updated. Detailed information about NBH-ADsnp is available in the Supplementary Material. This study has been approved by the Association for Responsible Human Participant Ethics Committee of the Affiliated Brain Hospital of Nanjing Medical University School (2018-KY010-01, 2020-KY010-42). All volunteers provided informed consent.

### Participants

Patients with SCD and healthy seniors were recruited from local communities through advertisements and broadcasts, while patients with aMCI were recruited from both the hospital and local communities. Initially, 234 individuals from the baseline period of the NBH-ADnsp database were enrolled. Among them, eight participants were excluded due to excessive head motion (cumulative translation or rotation of > 3.0 mm or 3.0°). According to previous studies (Wang et al., 2021; Xue et al., 2021), a total of 194 participants, including 59 aMCI, 57 SCD, and 78 HC, were eventually included after strict exclusion. Detailed information about inclusive and exclusive criteria is summarized in the Supplementary Material.

### Neuropsychological Assessment

A comprehensive neuropsychological assessment covering general cognitive function and four cognitive domains, including episodic memory (EM), executive function (EF), visuospatial function (VF), and information processing speed (IPS), was conducted among the participants (Chen et al., 2020; Wang et al., 2021; Xue et al., 2021; Xu et al., 2021). All evaluations were performed by two experienced neuropsychologists (Dr. Chen and Song). Details regarding the assessment can be obtained in the Supplementary Material.

### MRI Data Acquisition

The detailed parameters of image acquisition, including resting-state fMRI images and structural MRI images, are provided in the Supplementary Material. Scanning technologists and preprocessors were unaware of the clinical status of the participants.

### Preprocessing of Resting-State fMRI

All fMRI images were preprocessed by DPABI software and implemented in MATLAB2014a (Yan et al., 2016). First, 10 initial volumes were discarded to equilibrate brain signals, and the remaining voxels were subjected to slice time correction and realignment. Subsequently, structural images were segmented into gray matter, white matter, and cerebrospinal fluid partitions using the DARTEL technique (The gray matter would be used as a covariate in the subsequent statistical analysis; Ashburner, 2009). Then, all images were normalized to the Montreal Neurological Institute (MNI) EPI template and resampled at 3 × 3 × 3 mm voxels. After normalization, the Friston 24-parameter model, cerebrospinal fluid, white matter, and linear drift signals were all regressed. At the same time, head motion scrubbing regressors were also used with a threshold at FD_Jenkinson > 0.2 for bad time (Chao-Gan, 2010). Finally, the fMRI data were temporal band-pass filtered (0.01–0.08 Hz; Li et al., 2018a). Subjects who moved more than 3° of rotations or 3 mm of translation were excluded. For FCD calculation, we did not adopt spatial smoothing (Tomasi, 2012; Cheng et al., 2021). To analyze resting-state FC, all fMRI images were smoothed with a 6 × 6 × 6 mm FWHM Gaussian kernel (Chen et al., 2020).

### FCD Mapping

The voxel-wise FCD map for each participant was analyzed using the Neuroscience Information Toolbox (NIT, version 1.3, http://www.neuro.uestc.edu.cn/NIT.html; Dong et al., 2018), based on the protocol introduced by Tomasi and Volkow (Tomasi, 2010). FCD maps, mainly gFCD, lFCD, and lrFCD, are defined as the number of voxels with an FC strength (correlation coefficient) greater than 0.6, as determined by previous studies (Tomasi, 2010; Tomasi and Shokri-Kojori, 2016). The gFCD value was calculated by counting the total number of FC between a voxel and other voxels. A growing algorithm was used to obtain the lFCD. In this algorithm, given a voxel χ0, the FC between χ0 and the voxel χi adjacent to χ0 was calculated. If the FC strength was > 0.6, χi could be a neighbor of χ0. Additionally, the voxel χj, adjacent to χi, could also add to the list of χ0 neighbors if it is functionally connected to χ0 *via* a correlation coefficient > 0.6. This formula was repeated until no voxel could be added. The lFCD represented the number of all neighbors of χ0. In addition, the lrFCD map of each participant was obtained by removing lFCD from gFCD. Finally, for standardization, FCD maps were divided by the mean FCD value.

### Seed-Based Functional Connectivity Analysis

A resting-state FC analysis was performed to show direct functional couplings between brain areas with altered FCD maps. Specifically, we used each region showing significant alteration of FCD as a spherical region of interest (ROI), with the center corresponding to the peak voxel (radius = 6 mm), and then conducted a Pearson correlation analysis between the average time courses of ROI and whole-brain voxel under the gray matter mask of the whole brain. Finally, fisher's r - z transformation was performed on all FC maps for normality (Chen et al., 2021a).

### Statistical Analyses

Statistical Package for Social Sciences (SPSS) software, version 22.0 (IBM, Armonk, NY, USA), was used to perform statistical analyses that were not related to voxel computations. The χ^2^-test and the analysis of variance ANOVA were used to compare the differences in demographic and neuropsychological data among the CN, SCD, and aMCI groups. *Post-hoc* comparisons were further conducted with the Bonferroni correction (*p* < 0.05).

Using DPABI software, a one-way ANOVA was applied to compare the differences in gFCD, lFCD, and lrFCD across three groups with age, gender, years of education, and gray matter volumes as covariates. The nonparametric permutation test (1000 permutations) was applied, and the significance level was set to *p* < 0.01 with a cluster size > 150 voxels. *Post-hoc* comparisons, using the two-sample t-test with the results of ANOVA analyses as the mask, were conducted with age, gender, education level, and gray matter volumes as covariates. To strictly correct the results, a TFCE and the familywise error (FWE) were applied with a threshold of *p* < 0.05 and a cluster size > 50 voxels.

For the resting-state FC analysis, it is important to note that we only performed further FC analysis between groups with significantly different FCD values. That is, if the FCD value of a seed point differed between all three groups, we would apply ANOVA and a two-sample t-test to determine the FC changes between groups. However, if the FCD value of the seed point differed between two groups, we would perform a two-sample t-test to analyze the FC changes between only these two groups. All statistical analyses included age, gender, years of education, and gray matter volume as covariates.

The Pearson correlation analysis was performed to examine the correlation between the altered FCD, FCs, and cognitive domains after controlling age, gender, and education level using SPSS software. Additionally, also in SPSS software, ROC curves were analyzed based on substantially changed indicators between the patient and HC groups, such as FCD and FC, to assess their value in the distinction of pre-Alzheimer's spectrum. Each index's discriminating performance was assessed independently. Moreover, significantly altered indexes were combined using a binary logistic regression model, and the model's performance was also evaluated. Finally, we assessed the sensitivity and specificity of each biomarker individually and in combination.

## Results

### Demographic and Clinical Characteristics of Participants

We found no statistical discrepancy in age (*P* = 0.3435) between the three groups but found significant differences in gender (*P* = 0.0252) and education level (*P* = 0.0402). To avoid the impact of demographic differences on subsequent analyses, all statistical analyses included gender, age, and education level as covariates. As expected, general cognitive function scores (the Mini-Mental State Examination and the Montreal Cognitive Assessment test) of patients with aMCI showed significant decreases as compared to those of patients with SCD (*p* < 0.01) and HCs (*p* < 0.001). In teams of every cognitive domain, including EM, EF, IPS, and VF, patients with aMCI also had statistical deficits in comparison with HC (*p* < 0.05) and SCD (*p* < 0.05). Moreover, for the Subjective Cognitive Decline Questionnaire (SCD-Q), there were significant alterations between the three groups (*p* < 0.001). Details can be obtained in Table 1.

**Table 1:** Demographics and clinical characteristics of HC, SCD, and aMCI groups.

	**HC (*n* = 78)**	**SCD (*n* = 57)**	**aMCI (*n* = 59)**	***F*-values (χ^2^)**	***P*-values**
Age (years)	63.51 (6.79)	65.09 (7.66)	65.10 (7.81)	1.074	0.3435
Gender (F/M)	48/30	47/10	38/21	7.362	0.0252^*^
Education level (years)	12.31 (2.51)	11.74 (2.73)	11.09 (3.07)	3.267	0.0402^b^^*^
MMSE	28.59 (1.22)	28.05 (1.49)	27.10 (2.09)	7.166	0.0000^b^^***^/^c^^**^
MoCA	25.47 (2.41)	24.91 (2.07)	23.03 (3.11)	7.918	0.0000^b^^***^/^c^^***^
SCD-Q	3.64 (1.43)	6.31 (0.82)	5.03 (1.87)	23.907	0.0000^a^^***^/^b^^***^/^c^^***^
**Composite** ***Z*****-scores of each cognitive domain**					
Episodic memory	0.240 (0.502)	0.286 (0.482)	−0.593 (0.641)	23.756	0.0000^b^^***^/^c^^***^
Executive function	0.212 (0.528)	0.167 (0.539)	−0.442 (0.600)	23.138	0.0000^b^^***^/^c^^***^
Information processing speed	0.197 (0.750)	0.107 (0.692)	−0.364 (0.702)	24.969	0.0000^b^^***^/^c^^**^
Visuospatial function	0.165 (0.649)	0.084 (0.658)	−0.299 (0.975)	6.750	0.0000^b^^*^/^c^^*^

*Data are presented as mean (standard deviation). Values for age and education level derived from ANOVA; gender from χ-square test; all clinical characteristics from ANOVA with age, gender, and education level as covariates*.

*HC, healthy control; SCD, subjective cognitive decline; aMCI, amnestic mild cognitive impairment; MMSE, Mini-Mental State Examination; MoCA, the Montreal Cognitive Assessment test; SCD-Q, Subjective Cognitive Decline Questionnaire*.

a *Post-hoc analyses showed a significant group difference between SCD and HC*.

b *Post-hoc analyses showed a significant group difference between aMCI and HC*.

c *Post-hoc analyses showed a significant group difference between aMCI and SCD; Bonferroni correction was applied for multiple comparisons*.

* *p < 0.05;*

** *P < 0.01;*

*** *P < 0.001*.

### Functional Connectivity Density Analyses

The analyses of FCD showed that gFCD and lrFCD values were statistically different between the SCD and HC groups or between the SCD and aMCI groups, but lFCD values were not found to be significantly altered between the three groups. Details are available in Table 2.

**Table 2:** Global, local, and long-range FCD alterations across three groups.

**FCD**	**Cluster index**	**Cluster size (voxels)**	**Brain regions**	**Peak MNI coordinate (x, y, z)**	**Peak intensity (*t*-value)**
Global FCD	ANOVA				
	1	436	Left Parahippocampa Gyrus	−27,−27,−24	9.3845
	2	101	Right Temporal_Pole_Mid	45, 15,−42	7.7385
	SCD>HC				
	1	196	Left Parahippocampa Gyrus	−27,−27,−24	3.3817
	aMCI < SCD				
	1	121	Left Parahippocampa Gyrus	−27,−27,−24	−3.6892
Local FCD	NA				
Long-range FCD	ANOVA				
	1	686	Left Inferior Temporal Gyrus / Middle Frontal Gyrus / Hippocampus	−9,−6,−45	10.8675
	2	242	Right Temporal_Pole_Mid	18, 46, 12	7.8173
	SCD>HC				
	1	355	Left Hippocampus	−27,−18,−18	3.5523
	aMCI < SCD				
	1	301	Left Parahippocampa Gyrus	−27,−27,−24	−3.1684

*Cluster size >150 voxels in ANOVA analysis, p < 0.01; Cluster size >50 voxels in post hoc test, p < 0.05, TFCE-FWE corrected; All results had controlled the effects of age, gender, education level, and gray matter volumes*.

*FCD, functional connectivity density; aMCI, amnestic mild cognitive impairment; SCD, subjective cognitive decline; HC, healthy control*.

**Figure 1:**
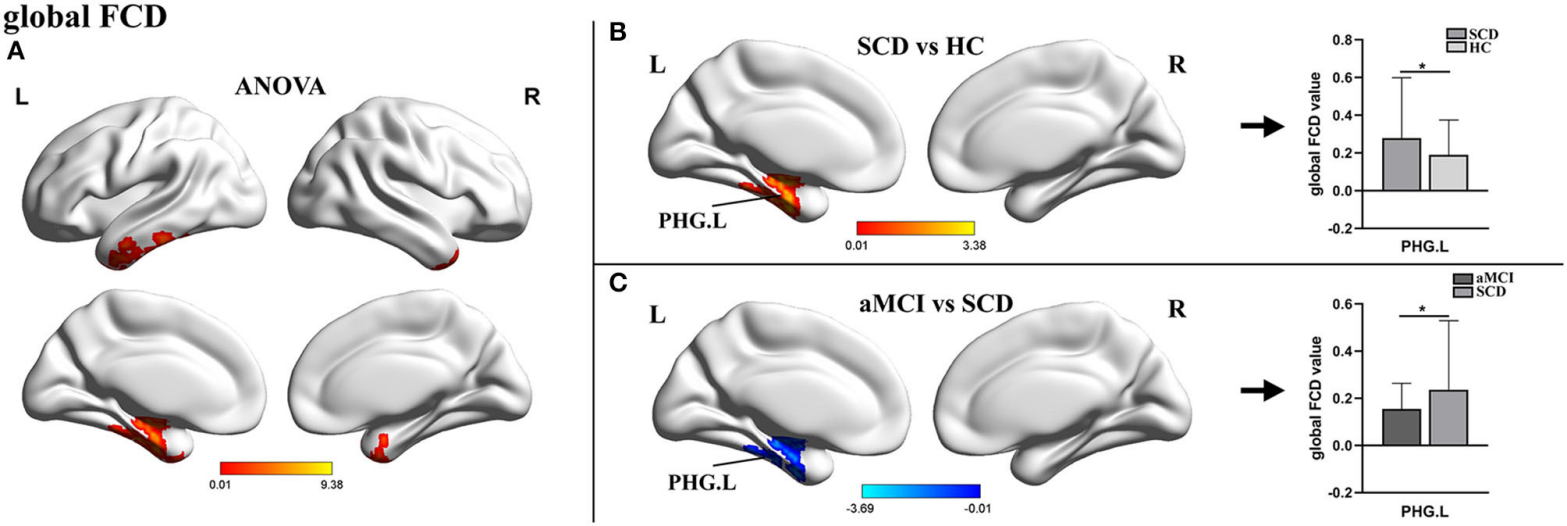
The distribution of brain regions with statistical differences in the global FCD for SCD, aMCI, and HC. **(A)** Shows the results of significant differences in global FCD across three groups (one-way ANOCA, *p* < 0.01, the cluster size >150 voxels). **(B,C)** Show the results of the *post-hoc* analysis across three groups (*TFCE-FWE corrected, *p* < 0.05, the cluster size >50 voxels). FCD, functional connectivity density; SCD, subjective cognitive decline; aMCI, amnestic mild cognitive impairment; HC, healthy control; PHG, parahippocampal gyrus; L, left; R, right.

For gFCD, the ANOVA analysis revealed significant differences between the left parahippocampal gyrus (PHG.L) and the right temporal pole: the middle temporal gyrus. In comparison to HC, the value of gFCD of the PHG.L was significantly different between groups, with significantly higher values in patients with SCD and significantly lower values in patients with aMCI (Table 2, Figure 1).

**Figure 2:**
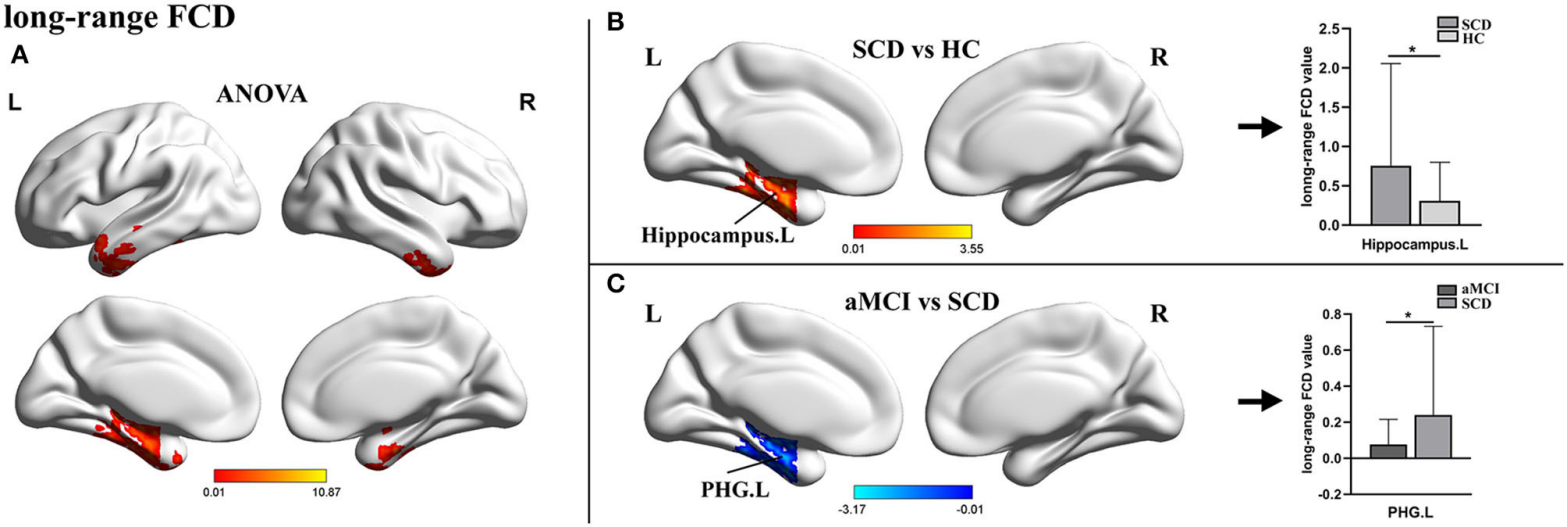
The distribution of brain regions with statistical differences in the long-range FCD for SCD, aMCI, and HC. **(A)** Shows the results of significant differences in long-range FCD across three groups (one-way ANOCA, *p* < 0.01, the cluster size >150 voxels). **(B,C)** Show the results of the *post-hoc* analysis across three groups (*TFCE-FWE corrected, *p* < 0.05, the cluster size >50 voxels). FCD, functional connectivity density; SCD, subjective cognitive decline; aMCI, amnestic mild cognitive impairment; HC, healthy control; PHG, parahippocampal gyrus; L, left; R, right.

For lrFCD, the ANOVA analysis revealed statistical differences in the left inferior temporal gyrus, the middle frontal gyrus (ITG.L/MFG.L), and the left hippocampus. In the *post-hoc* test, patients with SCD exhibited significantly higher lrFCD in the left hippocampus than HCs and significantly higher lrFCD in the PHG.L than patients with aMCI (Table 2, Figure 2).

### Functional Connectivity Analyses Based on Regions With Altered FCD

**Figure 3:**
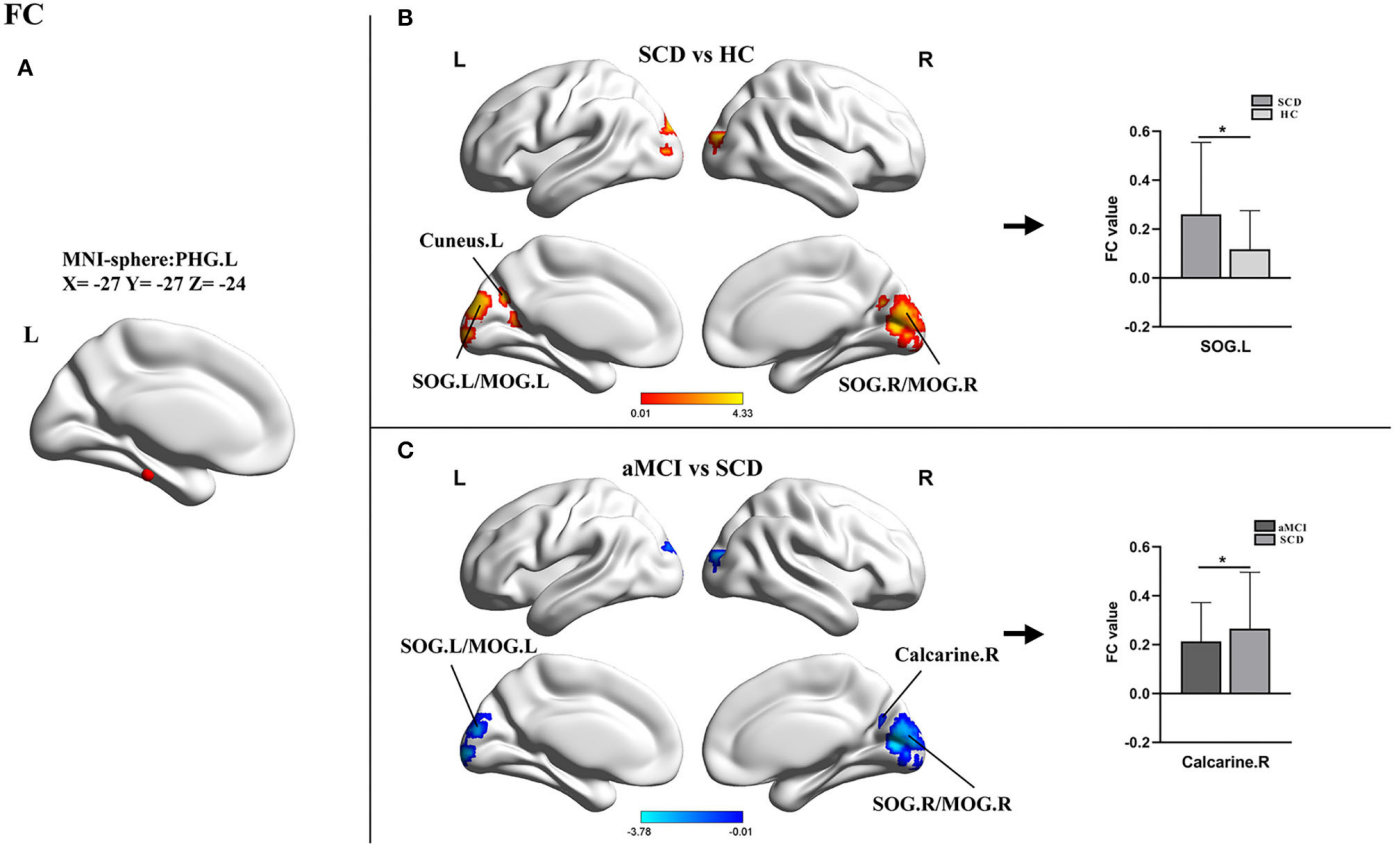
Results of the FC analyses with the PHG.L as a seed. **(A)** Shows the distribution of the seed. **(B,C)** Show results of the significant changes among groups (one-way ANOCA, TFCE-FEW corrected, *p* < 0.05, the cluster size >150 voxels; *post-hoc test, TFCE-FWE corrected *P* < 0.05, the cluster size > 50 voxels). FC, functional connectivity; SCD, subjective cognitive decline; aMCI, amnestic mild cognitive impairment; HC, healthy control; PHG, parahippocampal gyrus; SOG, superior occipital gyrus; MOG, middle occipital gyrus; L, left; R, right.

For the PHG.L seed (ROI 1, x = −27 y = −27 z = −24), the statistical differences in FCD values of this brain area involved three groups. An ANOVA analysis and a *post-hoc* test were carried out. Compared with HCs, patients with SCD showed markedly increased FC in bilateral superior occipital gyrus/middle occipital gyrus (SOG/MFG) and bilateral cuneus with the peak coordinate (x = −15, y = −87, z = 18) located in the left SOG (SOG.L). In addition, in comparison with SCD, the aMCI group presented significantly decreased FC in bilateral SOG/MFG and right calcarine with the peak coordinated (x = 6, y = −78, z = 9) located in the right calcarine. No statistical significance was found between aMCI and HC groups (Table 3, Figure 3).

**Table 3:** Regions with changed resting-state FC based on seed-based analyses across three groups.

**Seed (MNI-sphere)**	**Cluster index**	**Cluster size (voxels)**	**Brain regions**	**Peak MNI coordinate (x, y, z)**	**Peak intensity (*t*-value)**
ROI1 (x = −27, y = −27, z = −24)	ANOVA				
	1	799	Bilateral Superior Occipital Gyrus / Middle Occipital Gyrus / Cuneus	−15,−87, 18	10.3461
	SCD>HC^*^				
	1	799	Bilateral Superior Occipital Gyrus / Middle Occipital Gyrus / Cuneus	−15,−87, 18	4.3291
	aMCI < SCD^*^				
	1	492	Bilateral Superior Occipital Gyrus / Middle Occipital Gyrus / Right Calcarine	6,−78, 9	−3.7846
ROI2 (x = −27, y = −18, z = −18)	SCD>HC^**^				
	1	174	Left Cerebellum Anterior Lobe	−9,−51,−6	4.3587
	2	128	Left Inferior Temporal Gyrus	−48,−63,−6	4.3412

*When analyzing FC with ROI1 as the seed, the ANOVA analysis was carried out with TFCE-FWE corrected P < 0.05 and a cluster size > 150 voxels; in the post-hoc test, TFCE-FWE corrected*

* *P < 0.05 and a cluster size > 50 voxels were set. A voxel-wise two-sample t-test was applied when analyzing FC with ROI2 as the seed with TFCE-FWE corrected*

** *P < 0.005 and a cluster size >50 voxels. All results had controlled the effects of age, gender, education level, and gray matter volumes*.

*FC, functional connectivity; ROI, region of interest; SCD, subjective cognitive decline; HC, healthy control*.

**Figure 4:**
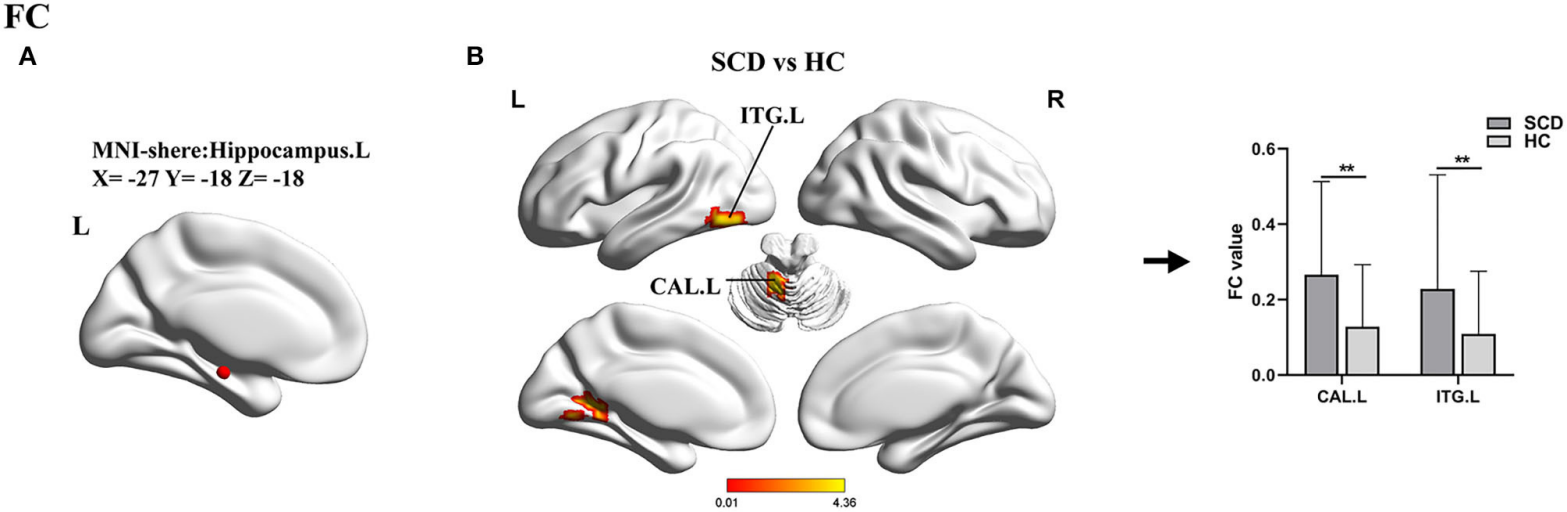
Results of the FC analyses with the hippocampus.L as a seed. **(A)** Shows the distribution of the seed. **(B)** Shows results of a two-sample *t*-test between SCD and HC (**TFCE-FEW corrected, *p* < 0.005, the cluster size >50 voxels). FC, functional connectivity; SCD, subjective cognitive decline; HC, healthy control; ITG, inferior temporal gyrus; CAL, cerebellum anterior lobe; L, left; R, right.

For the left hippocampus seed (ROI 2, X = − 27, Y = −18, Z = −18), a two-sample t-test was applied between SCD and HC groups. In this comparison, the SCD group exhibited increased FC in the left cerebellum anterior lobe (CAL.L) and the ITG.L (Table 3, Figure 4).

### Correlation Analyses

**Figure 5:**
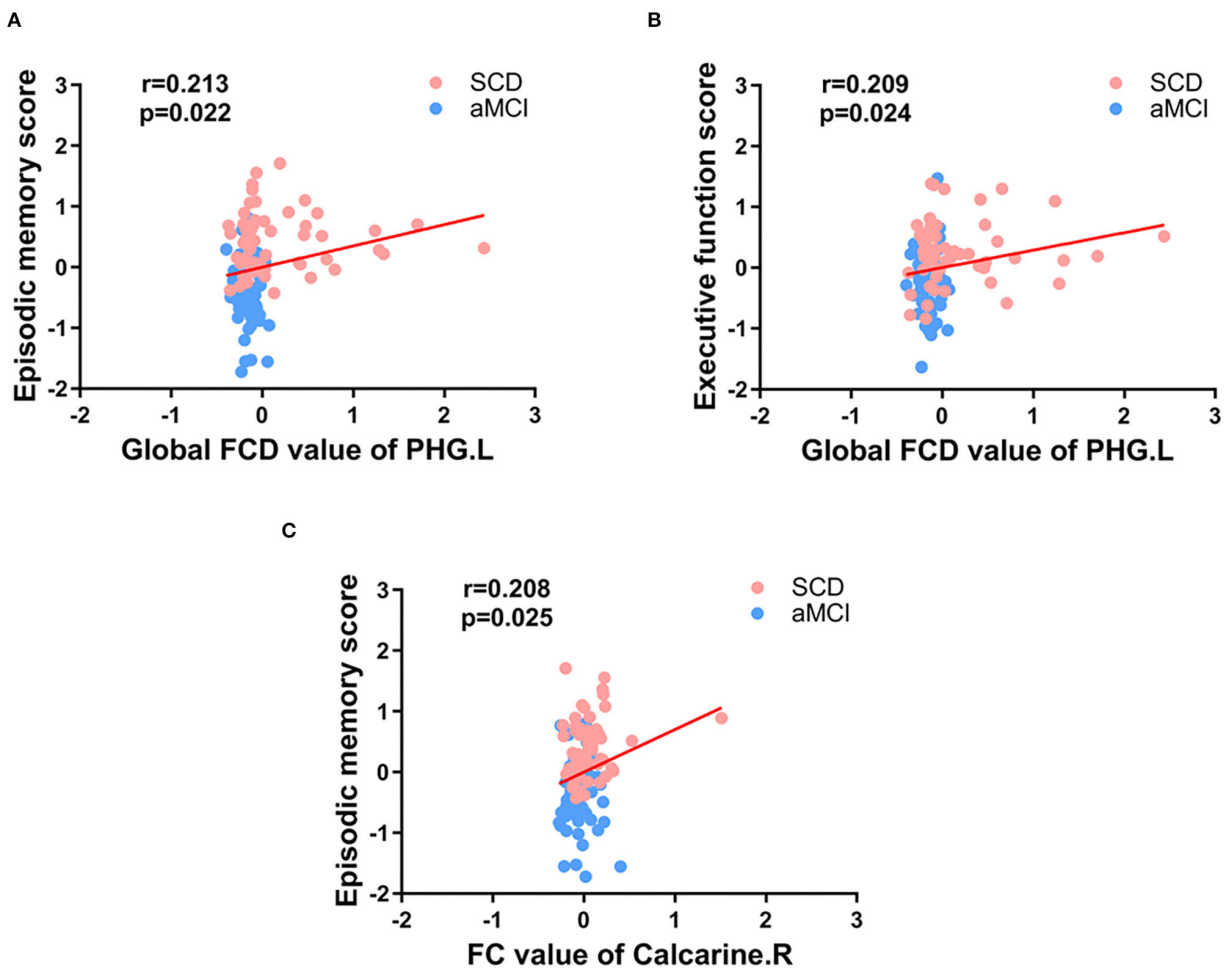
Significant associations between the FCD value or FC value in abnormal brain regions and cognitive function **(A-C)**. Age, gender, and education level were used as covariates (Bonferroni corrected, *p* < 0.05). SCD, subjective cognitive decline; aMCI, amnestic mild cognitive impairment; FC, functional connectivity; FCD, functional connectivity density; PHG, parahippocampal gyrus; L, left; R, right.

Pearson correlation analysis showed that, in the groups consisting of aMCI and SCD, the abnormal gFCD value in the PHG.L was prominently positively correlated with EM and EF. Besides, the altered FC between the PHG.L and right calcarine was also correlated with EM positively (Figure 5).

### Receiver-Operating Characteristic Analyses

**Figure 6:**
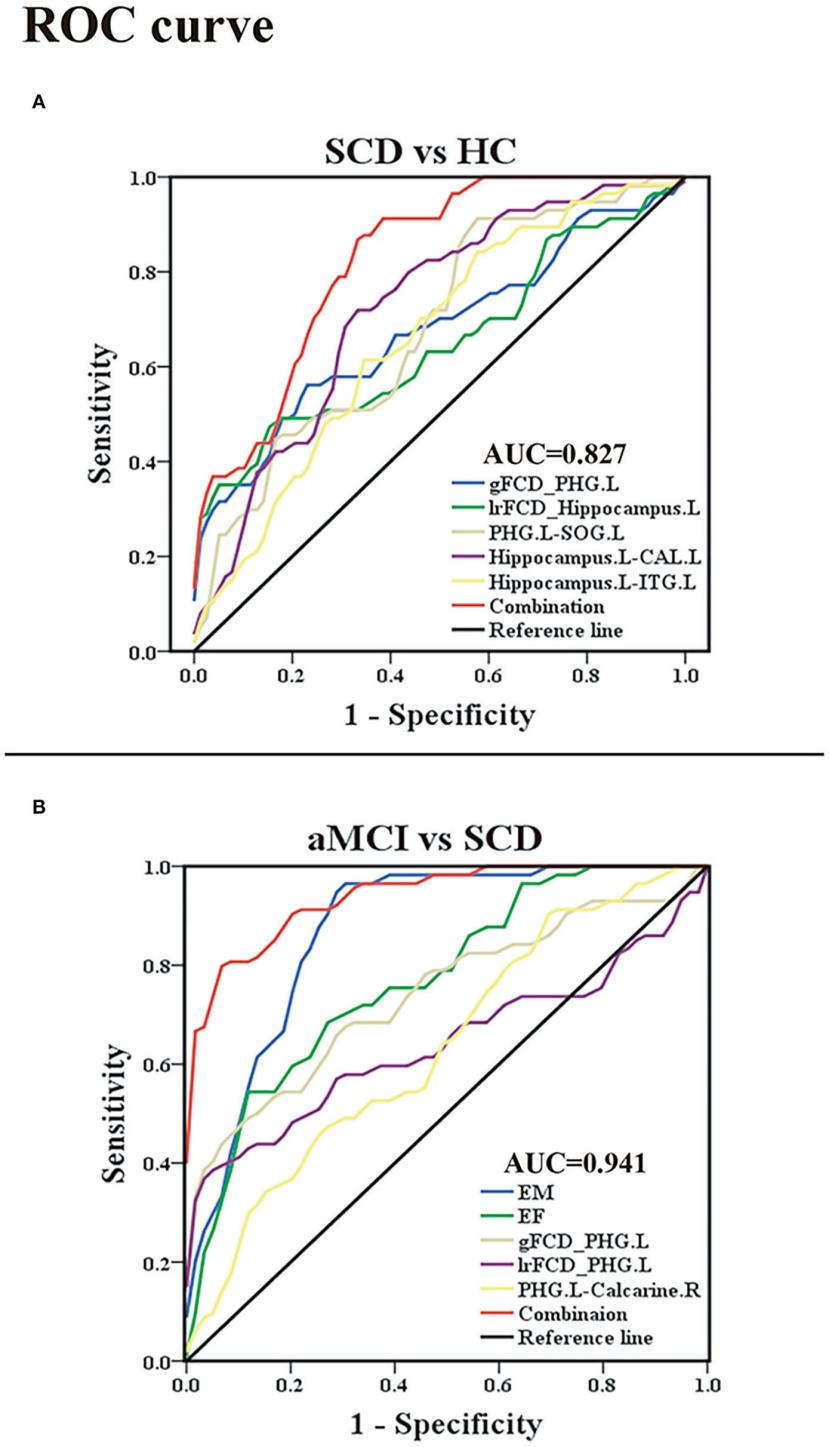
Classification of individuals as SCD vs. HC and aMCI vs. SCD based on the ROC analysis. **(A)** The ROC curve presenting the classification of SCD and HC; **(B)** ROC curve showcasing the classification of aMCI and SCD. ROC, receiver operating characteristic; AUC, area under the ROC curve (of the combination of multiple indexes); SCD, subjective cognitive decline; aMCI, amnestic mild cognitive impairment; HC, healthy control; gFCD, global functional connectivity density; lrFCD, long-range functional connectivity density; EM, episodic memory; EF, executive function; PHG, parahippocampal gyrus; SOG, superior occipital gyrus; ITG, inferior temporal gyrus; CAL, cerebellum anterior lobe; L, left; R, right.

Within the group containing SCD and HC, the area under the curve (AUC) values of gFCD of the PHG.L and lrFCD of the left hippocampus were 0.678 with *p* < 0.001 and 0.652 with *p* = 0.003. FC between PHG.L and SOG.L, FC between the left hippocampus and CAL.L, and FC between the left hippocampus and ITG.L had AUC values of 0.684 with *p* < 0.001, 0.722 with *p* < 0.001, and 0.661 with *p* = 0.001, respectively. In addition, using a binary logistic regression model, we obtained individual predictors of the combination of multiple indexes. The AUC value of the combination of multiple indexes was 0.827 with *p* < 0.001, sensitivity = 87.7%, specificity = 66.7% (Figure 6).

Within the group containing aMCI and SCD, the AUC values of the EM and EF were 0.870 with *p* < 0.001 and 0.771 with *p* < 0.001. The AUC values of gFCD of the PHG.L and lrFCD of the PHG.L were 0.741 with *p* < 0.001 and 0.643 with *p* < 0.08. The AUC value of FC between the PHG.L and right calcarine was 0.635 with *p* < 0.012. In addition, the AUC value of the combined index was 0.941 with *p* < 0.001, sensitivity = 93.2%, specificity = 80.7% (Figure 6).

## Discussion

This was the first study to examine the alterations in FCD maps and relevant functional connectivity in preclinical and early-stage AD and their clinical implications. According to our findings, in patients with SCD and aMCI, abnormalities in FCD emerged largely in the hippocampus and PHG. Based on FCD analyses, further FC analyses revealed that altered FC is mainly located in the occipital lobe, including the SOG, MOG, cuneus, and calcarine. Notably, the differences between MCI and SCD were linked to damages in cognitive domains. In addition, patients with SCD also had altered FC of the hippocampus with the cerebellum and the temporal lobe compared with HCs. In general, these findings have crucial implications for understanding the neural mechanisms that underpin these disparate manifestations of different stages of the preclinical AD spectrum.

### Altered FCD in SCD and aMCI

The significant changes in the gFCD between the three groups were exhibited in PHG. The PHG, bordering the subiculum and lying adjacent to the hippocampus, is the prime cortical input of the hippocampus and a crucial part involved in episodic memory encoding and recognition (van Strien and Cappaert, 2009; Mégevand et al., 2014). Although it is generally accepted that patients with SCD suffer from mild neuronal damage, sufficient functional compensation allows for regular clinical and cognitive performance. Here, we found that the gFCD of the PHG in SCD was much higher in HCs, which indicated that PHG established more efficient functional connections with other regions. This finding was consistent with previous research on topological changes in the functional connectome, which revealed that increased nodal properties such as global nodal efficiency and nodal strength in the SCD were primarily found in the default mode network (DMN), including the medial temporal and PHG (Li et al., 2018b; Chen et al., 2020a). However, in patients with aMCI, the gFCD of the PHG was markedly decreased compared with patients with SCD, a trend consistent with the previous studies (Liu et al., 2016; Mao et al., 2021; Yuan et al., 2021). This result suggested that, as the disease progressed, functional compensations became insufficient to compensate for further neurological damage, resulting in objective impairment in cognitive domains. Moreover, it has been confirmed that the presence of neurofibrillary tangles started in this brain area (Braak, 1991). Thus, we hypothesized that the absence of compensatory mechanisms was a manifestation of further impairment of neural functions in individuals.

In terms of the lrFCD, patients with aMCI also showed a decreased change in the PHG when compared with patients with SCD. Given that lrFCDs were derived from gFCD scores and that there were no significant changes in lFCDs, it was no surprise that analyses of the lrFCD and gFCD could obtain consistent results. However, compared with HCs, the abnormal lrFCD of patients with SCD was located in the hippocampus. The hippocampus, one of the most studied regions, plays an essential role in the creation of new memories, pattern separation, and cognitive flexibility (Virley et al., 1999; Sekeres et al., 2021). A mediation analysis revealed that indirect FCs between the hippocampus and other brain areas were mediated by the PHG (Ward et al., 2014), which may be one of the reasons for the different results of the two indicators. Existing rs-fMRI studies on patients with SCD have been more concerned with exploring the significance of general functional connectivity alterations (Liang et al., 2020; Xue et al., 2021), and this is the first report of specific alterations of the lrFCD in the hippocampus of patients with SCD. We hypothesized that this specific elevation was also a manifestation of functional compensation and could become a specific biomarker that distinguishes SCD from normal aging.

Reviewing the results of the FCD analyses, we found that FCD abnormalities (increased or decreased) were first found in the left hemisphere. It is well-known that directional hemispheric dominance has been established for a variety of cognitive functions and that lateralization is particularly important for cognitive functions (Banks et al., 2018; Vingerhoets, 2019). Available studies suggested that left functional lateralization of the DMN was influenced by aging and/or AD progression (Banks et al., 2018). Furthermore, in terms of structure, in patients with MCI and AD, brain atrophy was also left lateralized (Chen et al., 2020b). Therefore, we hypothesized that left functional lateralization in FCD of patients with SCD might have a more compensatory role, whereas a decrease in functional lateralization suggested further disease progression.

### Altered FC in SCD and aMCI

Using the left PHG as the seed, we found significantly altered FC in the occipital lobe, including SOG, MOG, cuneus, and calcarine in patients with SCD and aMCI and the changing trends were consistent with the results of FCD analyses. According to the processing theory of the memory system, the occipital-temporal visual object processing pathway is important for memory formation (Ren et al., 2019). Studies on scene perception revealed that the occipital place area, as well as the parahippocampal place area, played a key role in scene recognition and spatial perception (Julian et al., 2016; Epstein, 2019). Previous investigations also determined statistically increased spontaneous functional activity in the occipital lobe in SCD when compared with HCs (Sun et al., 2016). The SOG, MOG, and calcarine were considered to belong to the visual network (VN). Our results confirmed again that patients with SCD had increased connectivity in VN (Lista et al., 2015; Liang et al., 2021). However, there were some studies that reported disrupted FC of the PHG to other brain areas such as the posterior cingulate cortex when comparing patients with SCD and HCs (Chen et al., 2020; Sharma et al., 2021). In actuality, the PHG is a region consisting of five sub-regions, including the parasubiculum, the presubiculum, the perirhinal cortex, the entorhinal cortex, and the post rhinal cortex. Each subregion may develop different functions during cognitive impairment, and our results were only for a small part of the PHG (van Strien and Cappaert, 2009).

In addition, one study about functional brain networks reported internetwork connectivities between the DMN (PHG et al.) and the VN (Cai et al., 2017) when comparing patients with aMCI to HCs. The cuneus gradually becomes hypoperfused and develops amyloid deposits earlier in patients with prodromal AD due to pathological downregulation (Cho et al., 2013; Ding et al., 2014). Furthermore, acetylcholinesterase activity in the occipital lobe was found to be reduced in patients with MCI and positive AD biomarkers (Richter et al., 2019). Therefore, we hypothesized that the increased FC of the PHG to the occipital lobe was a compensatory response to mild cognitive decline, and this compensation gradually dissipated as neuronal damage increased.

Furthermore, using the left hippocampus as a seed, we found significantly increased FC in the CAL and ITG of patients with SCD when compared to healthy elders. Although motor coordination has traditionally been considered the cerebellum's core function, it is now well-understood that the cerebellum is also involved in learning and sensory processing, and the CAL was proved to be associated with visual-spatial and executive function (Schmahmann, 1998; Salman, 2016). The ITG is identified as a tertiary visual association cortex and is involved in the learning of cognitive tasks (Scheff et al., 2011). Experiments in mice have demonstrated that the senescence in the cerebellum was present earlier than that in the hippocampus (Woodruff-Pak et al., 2010). Previous studies reported that patients with SCD had increased cerebral blood flow in the hippocampus and ITG relative to HCs and patients with MCI (Thomas et al., 2021). All these results further confirmed that, despite the absence of objective cognitive impairment in SCD, reactive changes in brain function, especially functional compensation, had occurred.

### Behavioral and Clinical Significance of FCD and FC Abnormalities in SCD and aMCI

Correlation analyses showed that reduced gFCD values in the PHG were accompanied by decreased EM and EF in patients with aMCI and SCD, and FC values in the occipital lobe were also positively correlated with EM. These results further confirmed that the PHG, as well as the occipital lobe, were closely related to cognitive function, which was consistent with previous studies (Li et al., 2018b; Chen et al., 2020a). In contrast, in the SCD and HC groups, the results of the assessment of each cognitive domain were not significantly correlated with functional changes. Such a result was not unexpected, as there were no abnormalities in patients with SCD on the neuropsychological scales (Liang et al., 2020; Chen et al., 2021b). Because there are no effective and objective methods for diagnosing SCD or determining whether patients with SCD have been exacerbated, existing studies have attempted to improve disease differentiation accuracy by combining multiple indicators (Hadjichrysanthou et al., 2020; Parker et al., 2020; Mao et al., 2021). By ROC analysis, Xue et al. (2021) combined abnormal FC of the abnormal salience network, ALFF, and gray matter volume with the AUC value up to 0.852 in the SCD and HC groups and combined scale assessment results, cortical thickness of abnormal brain regions, and FC changes with the AUC value up to 0.931 in the SCD and aMCI groups. However, our study found that, in the SCD and HC groups, by virtue of changes in the FCD in the PHG and the hippocampus and altered FC, the AUC value was 0.827. Although the accuracy was not very high, the differentiation method was simpler. In addition, in the SCD and aMCI groups, the combination of scales, the FCD and FC changes in the PHG resulted in an AUC value of 0.941, which was more accurate. All results showed the advantages of FCD and FC analyses in studying preclinical and early-stage AD and provided a new vantage point for the diagnosis and differentiation of preclinical and early-stage AD, especially the conversion of SCD to aMCI.

Reviewing the study results, we did not find statistical differences between the aMCI and HC groups, which contradicted the general perception (Wang et al., 2013). In fact, previous studies based on FCD analyses were more centered on patients with AD and patients with MCI (Mao et al., 2021; Liu et al., 2022), whereas the current study was the first to include patients with aMCI and SCD, resulting in a dearth of comparable research. Second, FCD maps of patients with aMCI at different stages of aMCI may yield inconsistent results (Song et al., 2021; Liu et al., 2022). We hypothesized that, because most of the volunteers with aMCI in this study were in the early stage of the disease, the neurological damage was not very severe, and the decreased and compensatory elevation of FC among brain regions caused no significant changes in their FCD maps compared to HCs.

### Limitations

It is important to note that this study has several limitations. First, the relatively small sample size limited the conclusions that could be drawn. However, because our NBA-ADsnp database is constantly being updated, more participants will be included in future studies. Second, the gender and education levels of the three groups were not exactly matched, which might have affected our results. To avoid this effect, we included age, gender, and education as covariates in all statistical analyses. Third, in FCD analyses, we set a single threshold based on prior knowledge, and this fixed value could also lead to false positives or false negatives; thus, further studies will assess the between-group differences in FCD for different thresholds to better examine the stability of the results. Finally, this study was a cross-sectional study, and further analytical studies of longitudinal data are needed to help fully assess the neuroimaging changes in preclinical and early-stage AD.

## Conclusion

Our study investigated changes in FCD maps in the brain in SCD and aMCI. We found that the abnormalities of the FCD maps were predominantly localized in the PHG and the hippocampus, whereas FC alterations were mainly located in the occipital lobes, the cerebellum, and the ITG. In comparison to HCs, the changes in patients with SCD showed a specific increase, which may suggest the presence of compensatory mechanisms; in comparison to SCD, the changes in patients with aMCI showed a specific decrease, which was indicative of increasing impairment and closely related to cognitive decline. Furthermore, these abnormalities could be used to identify patients at different stages of the preclinical AD spectrum and may serve as potential biomarkers for diagnosis.

## Data Availability Statement

The raw data supporting the conclusions of this article will be made available by the authors, without undue reservation.

## Ethics Statement

The studies involving human participants were reviewed and approved by the Ethics Committees of the Affiliated Brain Hospital of Nanjing Medical University. The patients/participants provided their written informed consent to participate in this study. Written informed consent was obtained from the individual(s) for the publication of any potentially identifiable images or data included in this article.

## Author Contributions

XLin, JC, and YS designed the study. HW, SC, CX, YS, QY, HG, WQ, XLia, and ZY collected the data. YS, HW, and SC analyzed the data and prepared the manuscript. All authors contributed to the article and approved the submitted version.

## Funding

This study was supported by the National Natural Science Foundation of China (No. 81701675); the Key Project supported by Medical Science and the Technology Development Foundation, Nanjing Department of Health (No. JQX18005); and the Key Research and Development Plan (Social Development) Project of Jiangsu Province (No. BE2018608).

## Conflict of Interest

The authors declare that the research was conducted in the absence of any commercial or financial relationships that could be construed as a potential conflict of interest.

## Publisher's Note

All claims expressed in this article are solely those of the authors and do not necessarily represent those of their affiliated organizations, or those of the publisher, the editors and the reviewers. Any product that may be evaluated in this article, or claim that may be made by its manufacturer, is not guaranteed or endorsed by the publisher.
