# Supplementary material for: Differential Abnormality in Functional Connectivity Density in Preclinical and Early-Stage Alzheimer's Disease
Source: Front Aging Neurosci. 2022 May 25;14:879836. doi: 10.3389/fnagi.2022.879836 (PMC9177137; doi:10.3389/fnagi.2022.879836)
Supplement: Supplementary file 1 [file Data_Sheet_1.PDF]

Supporting Information

SI Methods

S.1 NBH-ADsnp database

S.2 Inclusive and exclusive criteria for participant

S.3 Neuropsychological assessments for the NBH-ADsnp database

S.4 Image acquisition for the NBH-ADsnp database

S.5 Image preprocessing

S.1 NBH-ADsnp database

Data used in this study were obtained from the Nanjing Brain Hospital-Alzheimer's Disease (AD) Spectrum Neuroimaging Project (NBH-ADsnp) database (in-home website: http://192.168.8.100) (Nanjing, China). NBH-ADsnp was derived from an AD Spectrum Neuroimaging Project that was launched in January 2018 by the Institute of Brain Functional Imaging, the Affiliated Brain Hospital of Nanjing Medical University (Nanjing, China). Prof. Jiu Chen, PhD, MD, from the Affiliated Brain Hospital of Nanjing Medical University, served as the principal investigator of NBH-ADsnp. NBH-ADsnp was initiated by Dr. Jiu Chen and Dr. Xiangrong Zhang and was named by Dr. Jiu Chen's research group (discussed by Chen Xue, Guanjie Hu, Wenwen Xu, Wan Liu, Wenzhang Qi, Siyu Wang, Jiani Xu, Shanshan Chen, and finally verified by Jiu Chen and Xiangrong Zhang). NBH-ADsnp is an observational study, which includes cross-sectional and longitudinal follow-up components. The goal of NBH-ADsnp was to identify early neuroimaging biomarkers of preclinical AD spectrum (Eliassen et al., 2017 and AD and AD), to predict disease progression of individuals within the preclinical AD spectrum, and to provide imaging-based targets for individualized intervention to prevent disease deterioration from preclinical stages to the eventually progressed AD. Initially, several hundreds of elderly individuals in NBH-ADsnp, who were all Han Chinese and right-handed, were recruited from hospitals and local communities by advertising and by means of broadcasting. This database used

a standardized clinical evaluation protocol that included a medical history interview, neurologic examination, a battery of neurocognitive assessments, and a resting-state MRI scan (T1, T2, 3D T1, DTI, and BOLD) for all participants (healthy controls (HC), SCD, naMCI, aMCI, and AD). All subjects and their study partners completed the informed consent process, and study protocols were reviewed and approved by the responsible Human Participants Ethics Committee of the Affiliated Brain Hospital of Nanjing Medical University (No. 2018-KY010-01, No. 2020-KY010-02, and No. ChiCTR1900022287).

S.2 Inclusive and exclusive criteria for participants

All volunteers participated voluntarily with written informed consent. The inclusive and exclusive criteria for participants were performed as described in our previous research (J. Chen et al., 2019; J. Chen, Shu, et al., 2016; Xue et al., 2019).

The inclusion criteria for the participants: (1) right-handed Chinese Han patients aged between 50 and 80 years old; (2) secondary school education or higher; (3) no history of taking psychoactive drug; (4) no history of serious brain diseases, such as brain tumor, brain infarction, brain hemorrhage, and severe brain injury; (5) no history of disease which could influence brain function, such as neuropathy, psychosis, thyroid dysfunction, and systemic disease; (6) no history of taking cognitive improvement drugs.

The inclusion criteria of HC: (1) no memory complaints; (2) normal cognitive performance matched with age and education level; (3) Clinical Dementia Rating (CDR) = 0.

The inclusion criteria of SCD based on the published SCD research criteria proposed by the Subjective Cognitive Decline Initiative (SCD-I): (1) always complained of memory problems; (2) Subjective Cognitive Decline Questionnaire (SCD-Q) > 5; (3) normal cognitive performance of age- and education-matched norms; (4) CDR = 0

The inclusion criteria of aMCI: (1) patients complained of memory impairment of at least 3 months or relatives confirmed that the memory impairment last for more than 3 months; (2) impaired objective memory performance based on the one of the following conditions: a, two neuropsychological tests in the episodic memory function that were within ≤1.0 standard deviation (SD) of age-adjusted norms; b, one neuropsychological test in the episodic memory function and the other tests in the other cognitive domain, including visual spatial function, executive function, and information processing speed were within ≤1.0 SD of age-adjusted norms; (3) normal overall cognitive function evidenced by CDR = 0.5, MMSE score ≥24, Activities of Daily Living assessment-20 (ADL-20) ≤ 23, MDRS-2 ≥ 120, Hamilton Depression Rating Scale (HAMD) ≤ 7; (4) no dementia.

The exclusion criteria: (1) a past history of stroke (modified Hachinski Ischemic Scale score >4), alcoholism, head injury, brain tumors, Parkinson's disease, epilepsy, encephalitis, major depression (excluded by HAMD), or other neurological or psychiatric illnesses (excluded by clinical assessment and case history); (2) major medical illness (e.g., cancer, anemia, thyroid dysfunction, syphilis, or HIV); (3) severe visual or hearing loss; (4) inability to complete neuropsychological tests or with a contraindication for MRI; and (5) T2-weighted MRI showing major changes in white matter (WM), infarction, or other lesions (two experienced radiologists analyzed the scans).

S.3 Neuropsychological Assessments

Neuropsychological assessments were performed as described in our previous research papers (J. Chen et al., 2019; J. Chen, Duan, et al., 2016; J. Chen et al., 2015; J. Chen, Shu, et al., 2020; Xue et al., 2019). All subjects had a standardized clinical interview and underwent comprehensive neuropsychological assessments by 3 neuropsychologists (Dr. Song, Yuan, and Chen). The evaluation included the Mini Mental State Examination (MMSE), Montreal Cognitive Assessment (MoCA), Mattis Dementia Rating Scale (MDRS), Auditory Verbal Learning Test - immediate recall (AVLT-IR), Auditory Verbal Learning Test -5-min delayed recall (AVLT-5-min-DR), Auditory Verbal Learning Test-20-min delayed recall (AVLT-20-min-DR), Auditory Verbal Memory Test recognition (AVLT-R), Logical Memory Test-immediate recall (LMT-IR), Logical Memory Test-20-min delayed recall (LMT-20-min-DR), Rey-Osterrieth Complex Figure Test -20-min delayed recall (ROCFT-20min-DR), Clock Drawing Test (CDT), Rey-Osterrieth Complex Figure Test (ROCFT), Verbal Fluency Test (VFT)(including the VFT-animals and the VFT-objects), Digit Span Test (DST), Digital Symbol Substitution Test (DSST), Trail-Making Tests A and B (TMT-A and B), Stroop Color and Word Test A, B, and C, and Semantic Similarity (Similarity) test. AVLT-20-min DR, AVLT-R, LMT-20-min DR, and CFT-20-min DR constitute the episodic memory score. DSST, TMT-A, Stroop A, and Stroop B make up the information processing speed score. CFT and CDT form visuospatial function score. VFT, DST-backward, TMT-B, Stroop C, and Semantic Similarity constitute executive function score. These tests were used to evaluate general cognitive function, episodic memory, information processing speed, executive function, and visuo-spatial function.

S.4 MRI Data Acquisition

The NBH-ADsnp data acquisition process was also recorded in our former articles(J. Chen et al., 2019; J. Chen, Shu, et al., 2016; Xue et al., 2019). The acquisition of magnetic resonance imaging (MRI) data is all obtained through using a 3.0 Tesla Verio Siemens scanner with an 8-channel head-coil in the Affiliated Brain Hospital of Nanjing Medical University. The subjects were asked to close their eyes, stop thinking and remain still to ensure accurate resting state images. 240 volumes were included in the the echo-planar imaging (EPI) sequence. The

parameters were: repetition time (TR) = 2,000 ms, echo time (TE) = 30 ms, number of slices = 36, thickness = 4.0 mm, gap = 0 mm, matrix = 64 × 64, flip angle (FA) = 90°, field of view (FOV) = 220 mm × 220 mm, acquisition bandwidth = 100 kHz, and voxel size = 3.4 × 3.4 × 4 mm^3. The imaging took approximately 8 minutes.

High-resolution T1-weighted images were acquired by 3D magnetization-prepared rapid gradient-echo (MPRAGE) sequence, whose parameters were as follows: TR = 1,900 ms, TE = 2.48 ms, inversion time (TI) = 900 ms, number of slices = 176, thickness = 1.0 mm, gap = 0.5 mm, matrix = 256 × 256, FA = 9°, FOV = 256 mm × 256 mm, and voxel size = 1 × 1 × 1 mm^3. The imaging took approximately 4.26 minutes.

Routine axial T2-weighted images were acquired to rule out subjects with major changes in white matter (WM), cerebral infarction or other lesions using flair sequence as follows: TR = 8400 ms, TE = 94 ms, FA= 150°, acquisition matrix = 256×256, FOV = 230×230 mm, thickness = 5.0 mm, gap = 0 mm, and number of slices = 20. The imaging processtook approximately 2.50 minutes to complete.

S.5 Image preprocessing

All fMRI data were preprocessed using MATLAB 2014a (http://www.mathworks.com/products/matlab/) and DPABI image processing software(S. Chen et al., 2021; Yan, Wang, Zuo, & Zang, 2016). To maintain the stability of the MRI signal, the first 10 volumes were removed. Slice timing and motion effects were first corrected. Corrections were performed for the intra-volume acquisition time differences among slices and inter-volume motion effects during the scan(Power, Barnes, Snyder, Schlaggar, & Petersen, 2012; Van Dijk, Sabuncu, & Buckner, 2012). Structural images were then segmented into gray matter (Note: gray matter was used as a covariate in statistical comparison between groups), white matter and CSF partitions using the DARTEL technique(Ashburner & Friston, 2009). The fMRI data were spatially normalized to a standard EPI template and were resampled to 3×3×3 mm^3 voxels(J. Chen, Shu, et al., 2016). A Friston 24-parameter model was used to regress out head motion effects from the realigned data(Friston, Williams, Howard, Frackowiak, & Turner, 1996). CSF, white matter, as well as the linear trend were also regressed as nuisance covariates(Brady et al., 2019). After realigning, slice-timing correction, and co-registration, framewise displacement (FD) was calculated for all resting-state volumes(Power et al., 2012). All volumes with a FD greater than 0.2

mm were regressed out as nuisance covariates(Brady et al., 2019). Finally, Temporal band-pass filtering (0.01–0.1 Hz) was applied to reduce the effect of low-frequency drifts and high-frequency physiological noise. Voxels within a group GM mask, created by DARTEL, were used for further analyses(Yan, Craddock, Zuo, Zang, & Milham, 2013; Zhou et al., 2019). For FCD calculation, we didn't adopt spatial smooth(Cheng et al., 2021; Tomasi & Volkow, 2012). For analysis of resting-state FC, all fMRI images were smoothed with a 6 mm × 6 mm × 6 mm FWHM Gaussian kernel(J. Chen, Ma, et al., 2020)

Reference

Ashburner, J., & Friston, K. J. (2009). Computing average shaped tissue probability templates. *Neuroimage, 45*(2), 333-341. doi:10.1016/j.neuroimage.2008.12.008

Brady, R. O., Jr., Gonsalvez, I., Lee, I., Öngür, D., Seidman, L. J., Schmahmann, J. D., . . . Halko, M. A. (2019). Cerebellar-Prefrontal Network Connectivity and Negative Symptoms in Schizophrenia. *Am J Psychiatry, 176*(7), 512-520. doi:10.1176/appi.ajp.2018.18040429

Chen, J., Chen, G., Shu, H., Chen, G., Ward, B. D., Wang, Z., . . . Zhang, Z. (2019). Predicting progression from mild cognitive impairment to Alzheimer's disease on an individual subject basis by applying the CARE index across different independent cohorts. *Aging (Albany NY), 11*(8), 2185-2201. doi:10.18632/aging.101883

Chen, J., Duan, X., Shu, H., Wang, Z., Long, Z., Liu, D., . . . Zhang, Z. (2016). Differential contributions of subregions of medial temporal lobe to memory system in amnestic mild cognitive impairment: insights from fMRI study. *Sci Rep, 6*, 26148. doi:10.1038/srep26148

Chen, J., Ma, N., Hu, G., Nousayhah, A., Xue, C., Qi, W., . . . Zhang, X. (2020). rTMS modulates precuneus-hippocampal subregion circuit in patients with subjective cognitive decline. *Aging (Albany NY), 13*(1), 1314-1331. doi:10.18632/aging.202313

Chen, J., Shu, H., Wang, Z., Liu, D., Shi, Y., Zhang, X., & Zhang, Z. (2015). The interaction of APOE genotype by age in amnestic mild cognitive impairment: a voxel-based morphometric study. *J Alzheimers Dis, 43*(2), 657-668. doi:10.3233/jad-141677

Chen, J., Shu, H., Wang, Z., Zhan, Y., Liu, D., Liao, W., . . . Zhang, Z. (2016). Convergent and

divergent intranetwork and internetwork connectivity patterns in patients with remitted late-life depression and amnestic mild cognitive impairment. *Cortex, 83*, 194-211. doi:10.1016/j.cortex.2016.08.001

Chen, J., Shu, H., Wang, Z., Zhan, Y., Liu, D., Liu, Y., & Zhang, Z. (2020). Intrinsic connectivity identifies the sensory-motor network as a main cross-network between remitted late-life depression- and amnestic mild cognitive impairment-targeted networks. *Brain Imaging Behav, 14*(4), 1130-1142. doi:10.1007/s11682-019-00098-4

Chen, S., Song, Y., Wu, H., Ge, H., Qi, W., Xi, Y., . . . Chen, J. (2021). Hyperconnectivity Associated with Anosognosia Accelerating Clinical Progression in Amnestic Mild Cognitive Impairment. *ACS Chem Neurosci*. doi:10.1021/acschemneuro.1c00595

Cheng, B., Zhou, Y., Kwok, V. P. Y., Li, Y., Wang, S., Zhao, Y., . . . Wang, J. (2021). Altered Functional Connectivity Density and Couplings in Postpartum Depression with and Without Anxiety. *Soc Cogn Affect Neurosci*. doi:10.1093/scan/nsab127

Eliassen, C. F., Reinvang, I., Selnes, P., Grambaite, R., Fladby, T., & Hessen, E. (2017). Biomarkers in subtypes of mild cognitive impairment and subjective cognitive decline. *Brain Behav, 7*(9), e00776. doi:10.1002/brb3.776

Friston, K. J., Williams, S., Howard, R., Frackowiak, R. S., & Turner, R. (1996). Movement-related effects in fMRI time-series. *Magn Reson Med, 35*(3), 346-355. doi:10.1002/mrm.1910350312

Power, J. D., Barnes, K. A., Snyder, A. Z., Schlaggar, B. L., & Petersen, S. E. (2012). Spurious but systematic correlations in functional connectivity MRI networks arise from subject motion. *Neuroimage, 59*(3), 2142-2154. doi:10.1016/j.neuroimage.2011.10.018

Tomasi, D., & Volkow, N. D. (2012). Resting functional connectivity of language networks: characterization and reproducibility. *Mol Psychiatry, 17*(8), 841-854. doi:10.1038/mp.2011.177

Van Dijk, K. R., Sabuncu, M. R., & Buckner, R. L. (2012). The influence of head motion on intrinsic functional connectivity MRI. *Neuroimage, 59*(1), 431-438. doi:10.1016/j.neuroimage.2011.07.044

Xue, C., Yuan, B., Yue, Y., Xu, J., Wang, S., Wu, M., . . . Chen, J. (2019). Distinct Disruptive Patterns of Default Mode Subnetwork Connectivity Across the Spectrum of Preclinical

Alzheimer's Disease. *Front Aging Neurosci, 11*, 307. doi:10.3389/fnagi.2019.00307

Yan, C. G., Craddock, R. C., Zuo, X. N., Zang, Y. F., & Milham, M. P. (2013). Standardizing the intrinsic brain: towards robust measurement of inter-individual variation in 1000 functional connectomes. *Neuroimage, 80,* 246-262. doi:10.1016/j.neuroimage.2013.04.081

Yan, C. G., Wang, X. D., Zuo, X. N., & Zang, Y. F. (2016). DPABI: Data Processing & Analysis for (Resting-State) Brain Imaging. *Neuroinformatics, 14*(3), 339-351. doi:10.1007/s12021-016-9299-4

Zhou, C., Yu, M., Tang, X., Wang, X., Zhang, X., Zhang, X., & Chen, J. (2019). Convergent and divergent altered patterns of default mode network in deficit and non-deficit schizophrenia. *Prog Neuropsychopharmacol Biol Psychiatry, 89*, 427-434. doi:10.1016/j.pnpbp.2018.10.012
